# Diagnostic accuracy of MRI with MRCP and B-Mode-sonography with elastography of the pancreas in patients with cystic fibrosis: a point-to-point comparison

**DOI:** 10.1186/s13104-019-4193-4

**Published:** 2019-03-18

**Authors:** Christopher Kloth, Dorit Fabricius, Inka Wendlik, Stefan Andreas Schmidt, Matthias Pfahler, Elisabeth Lormes, Meinrad Beer, Wolfgang Kratzer, Julian Schmidberger

**Affiliations:** 1 Department of Diagnostic and Interventional Radiology, University Hospital Ulm, Albert-Einstein-Allee 23, 89081 Ulm, Germany; 2 Department of Paediatric and Adolescent Medicine, Ulm University Hospitals, Eythstrasse 24, 89075 Ulm, Germany; 3 Department of Internal Medicine I, University Hospital Ulm, Albert-Einstein-Allee 23, 89081 Ulm, Germany; 4 Department of Dermatology and Allergic Diseases, Ulm University Hospital, Albert-Einstein-Allee 23, 89081 Ulm, Germany

**Keywords:** Cystic fibrosis, Pancreas, MRI imaging, B-mode sonography, Shear wave elastography

## Abstract

**Objective:**

For patients with cystic fibrosis, the imaging of the pancreas is of crucial importance for the early detection of pancreatic carcinoma. Comparative studies between Magnetic Resonance Imaging (MRI) and sonographic pancreas sonography are not yet available. The aim of the study was to compare MRI, sonography and point-shearwave elastography (pSWE). A total of 19 patients were included (10 male, 9 female; age 29.7 ± 14.3 years) in the study. Ultrasonography with pSWE and contrast enhanced MRI with MRCP were performed.

**Results:**

Significant differences between measurements of pancreatic body were registered in MRI with 1.4 ± 0.6 cm vs 1.0 ± 0.4 cm in ultrasound (p = 0.049), however not for pancreatic head and tail. In 10/19 patients (52.6%) pancreatic parenchyma did not show in MRI because of complete lipomatous transformation, but could be detected in ultrasound. pSWE-values showed no significant differences between the full and partial fatty transformation in pancreatic head (p = 0.968), body (p = 0.657) and tail (p = 0.840). pSWE-values did not correlate with measured signal intensity in T1w flash (p = 0.930, r = 0.025) and T2w HASTE sequences (p = 0.152, r = − 0.375). In patients with CF ultrasound is superior to MRI for displaying full fibro-fatty parenchymal transformation, pancreatic duct. Ultrasound elastography did not provide additional clinical relevant information.

**Electronic supplementary material:**

The online version of this article (10.1186/s13104-019-4193-4) contains supplementary material, which is available to authorized users.

## Introduction

Patients with cystic fibrosis (CF) show a large spectrum of radiological findings concerning pancreatic parenchymal changes with replacement by fibrofatty tissue and lipomatous hypertrophy up to different forms of cystic transformation. Morphological imaging of pancreas is necessary to evaluate the status of the organ and the progression of the disease [1, 2]. Patients with CF need a sufficient imaging and screening follow-up, however the decision for right imaging method is challenging. For many years computed tomography therefore played an important role. This is a dilemma as the cancer risk increases with cumulative radiation dose, especially in patients with infantile and juvenile CF [3]. Due to the intensified antibiotic therapy, many cystic fibrosis patients reach adulthood, so that an increased risk of pancreatic carcinoma with increasing age can be assumed. The data situation remains contradictory [4, 5].

In this constellation imaging methods without radiation exposure should be chosen. Beside conventional ultrasonography also additional sonography methods like point shear wave elastography (pSWE) and MRI imaging, especially MR cholangiopancreatography (MRCP) may be eligible for this group of patients, both without radiation exposure [6, 7].

Comparison both imaging methods revealed a high consensus of results in current literature, however there also exist cases of discrepancy sonographic hyperechogenicity which may reflect fatty infiltration or fibrosis [8, 9]. In this context the diagnostic benefit of point shear wave elastography for fibrosis evaluation is still not finally investigated [10].

Aim of our single-centre study was to compare the diagnostic imaging methods of MRI and sonography, especially pSWE of the pancreas in patients with CF. We also aimed to compare both methods concerning morphological aspect of the pancreas like size, duct anatomy and detection of potential cystic lesions.

## Main text

### Patients and methods

The study cohort consisted of 19 patients (9 female; 10 male; mean age 29.7 ± 14.3 years; range 9–58) with CF. All patients received conventional b-mode ultrasonography, pSWE ultrasonography and native and contrast enhanced MRI sequences including diffusion weighted imaging (DWI) and MR-cholangiopancreatography (MRCP). Patients were recruited prospective from the cystic fibrosis outpatient clinic at the University Children’s Hospital. Precondition for inclusion in the study was CF according to the criteria of the guideline “diagnosis of cystic fibrosis” [11] and confirmed by prior genetic testing. Furthermore, patients had to fulfill the inclusion criteria of at least one MRI of the abdomen and ultrasonography of the pancreas. Patients with more than 80 days between MRI and ultrasound were excluded from the prospective study. Further exclusion criteria were pregnancy or unstable health status, which does not allow ultrasound examination. Six patients were excluded from final analysis because of missing MRI examination. Two patients were excluded from the final analysis because of a time difference of more than 80 days between MRI and ultrasound examination.

#### B-mode imaging

The examinations were performed by two advanced physicians with more than 5 years experience and more than 4000 examinations per year in ultrasonography using an ACUSON S3000 scanner (Siemens Healthineers, Erlangen, Germany). A fasting period of at least 3 h before ultrasonography examination was adhered. All patients underwent conventional B-mode ultrasound scans of the abdomen. A 6C1 HD transducer at 1.5 to 6.0 MHz was used for the B-Mode ultrasound and elastography. Diagnosis of pancreatic lipomatosis was made when the pancreas was significantly more echogenic than the adjacent liver parenchyma [12]. The pancreas was measured standardized at the head, body, and tail in concordance to current literature [10, 13]. The entire pancreas was depicted in a cross section of the upper abdomen. With the help of transversal sections it was possible to measure all sections (caput, corpus, cauda) of the organ in 3 different images. In each image, both the length (transverse diameter) and the depth (anterior–posterior diameter) of the respective organ section were measured. The confluence of the lienal vein and the superior mesenteric vein was defined as a point of orientation for delimiting the transition from caput to corpus, where the caput lies ventrally to the superior mesenteric vein and the corpus ventrally to the lienal vein. The cauda was determined as the anterior structure of the left kidney with expansion into the splenic hilus. At the point of intersection of the length and depth measurements in the cross-section, the transducer was rotated by 90° and thus a sagittal section of the respective pancreatic section was set, in which the width could be measured as the cranio-caudal diameter. In order to obtain the best possible approximation to the actual organ volume, the following modified ellipsoid formula was applied, which is frequently used in sonography for reproducible volume determination:

$${\text{Volume }}\left( {\text{in ml}} \right)\, = \,{{({\text{D1}} \times {\text{D2}} \times {\text{D3}})} \mathord{\left/ {\vphantom {{({\text{D1}} \times {\text{D2}} \times {\text{D3}})} 2}} \right. \kern-0pt} 2}$$

D1 corresponded to the length, D2 to the depth and D3 to the width. This formula was applied to all three sections of the pancreas, resulting in three partial volumes, which, when added together, gave the total volume. This approach had the advantage that the individual partial volumes could also be set in relation to each other. The total volume was finally set in relation to the body surface in order to obtain a comparable index (PVI). In addition to the volume measurement, the representation of the pancreas in the sonographic B-scan was also evaluated. Particular attention was paid to the echogenicity and homogeneity of the pancreatic parenchyma, the general shape of the organ, focal changes and the presentation of the pancreatic duct, whose lumen width is 1–2 mm in normal findings.

#### pSWE imaging

**Fig. 1:**
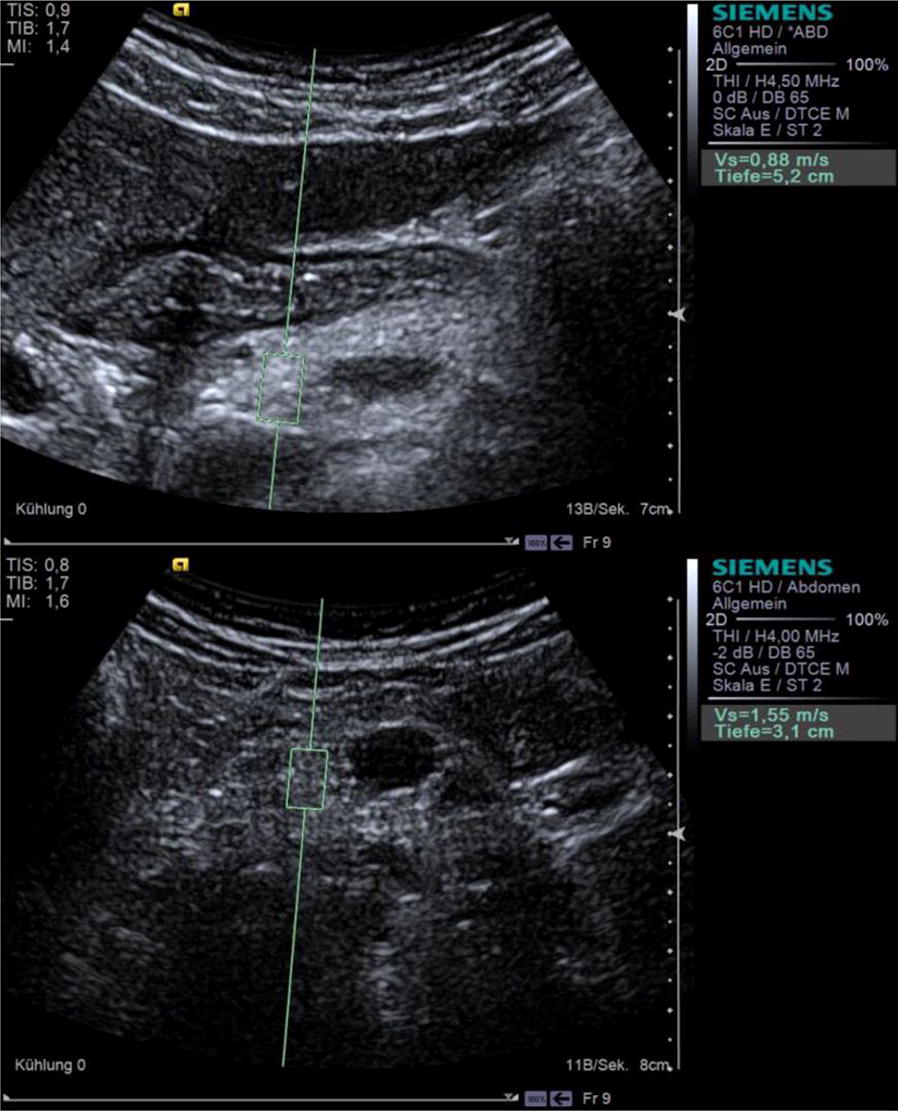
Shear wave velocities in the head of the pancreas. Measurement of shear wave velocities in the head of the pancreas in a patient with cystic fibrosis (top) and in a healthy control (bottom) by the VTQ mode

Elasticity of the pancreas was measured by virtual touch quantification (VTQ) in pSWE using the 6C1 HD convex transducer (Fig. 1) [10, 17]. This involves placing an approximately 10, 9, 6 mm measurement window (ROI) over the area of tissue to be analyzed. The result of the shear wave velocity is given in units of meters per second (m/s). In each case, we took four measurements in each of the head, body, and tail regions. To obtain measurements in mid-respiration, the subject was instructed to breathe in deeply, breathe out, and then breathe normally, until told to stop breathing while the measurements were taken. This procedure prevented motion artifacts, as far as possible.

#### MR imaging

MRI examinations were performed on a 3 T whole-body MRI system (Siemens MAGNETOM^®^ Skyra 3 T, Siemens Healthineers, Erlangen, Germany). All sequences were acquired in breath-hold technique. The examination protocol included standardized imaging protocols including precontrast T1-GRE-2D transversal, T1-GRE-2D fatsat coronal and transversal after contrast injection (0.1 mmol/kg of body weight of gadobutrol; Bayer Schering Pharma AG, Berlin-Wedding, Germany), diffusions weighted sequences with ADC-maps, T2-HASTE coronal und transversal. For MRCP, T2-RARE/T2-HASTE/MIP-HASTE coronal over corpus, head and tail and T2-Space3D coronal and dynamic MIP-MRCP sequences were acquired.

#### Image analysis

Size measurements of pancreatic head, body and tail, visibility of pancreatic duct, diameter of pancreatic duct, ascites, cystic lesions (single or multiple, size), edema, malignoma, concrement and inflammation were evaluated by both imaging methods. Signal intensities (SI) of the pancreatic head were evaluated in T1w-GRE and T2 HASTE sequences. Additionally, SI values were assessed in liver and muscle parenchyma and SI ratios calculated.

#### Statistical analysis

Statistical analyses were performed using SPSS Version 23.0 from IBM, Armonk, USA. The Kolmogorov-Smirnov test was used for the normality test including Lilliefors significance correction. Wilcoxon-rank test was used for comparing size of pancreatic head, body and tail. McNemar test was used for inflammation, representability of pancreatic duct and identification of cysts in both imaging methods. p-values testing for differences between subgroups were calculated. For calculation of correlations spearmans coefficient was calculated. A value of p < 0.05 was considered as statistically significant.

### Results

The study cohort consisted of 19 patients (9 female; 10 male; mean age 29.7 ± 14.3 years; range 9–58) with CF. All patients included in the study had an exocrine pancreatic insufficiency and a body-mass-index (BMI) less than 25.6 (31.6%) of the patients were under 18 years and 5 (26.3%) older than 40 years (19.85 ± 2.44). The mean age of the evaluated patients (n = 19) at diagnosis was 29.7 ± 14.3 years. A total of 31.5% (n = 6) patients had an endocrine pancreatic insufficiency.

#### MRI examination

**Fig. 2:**
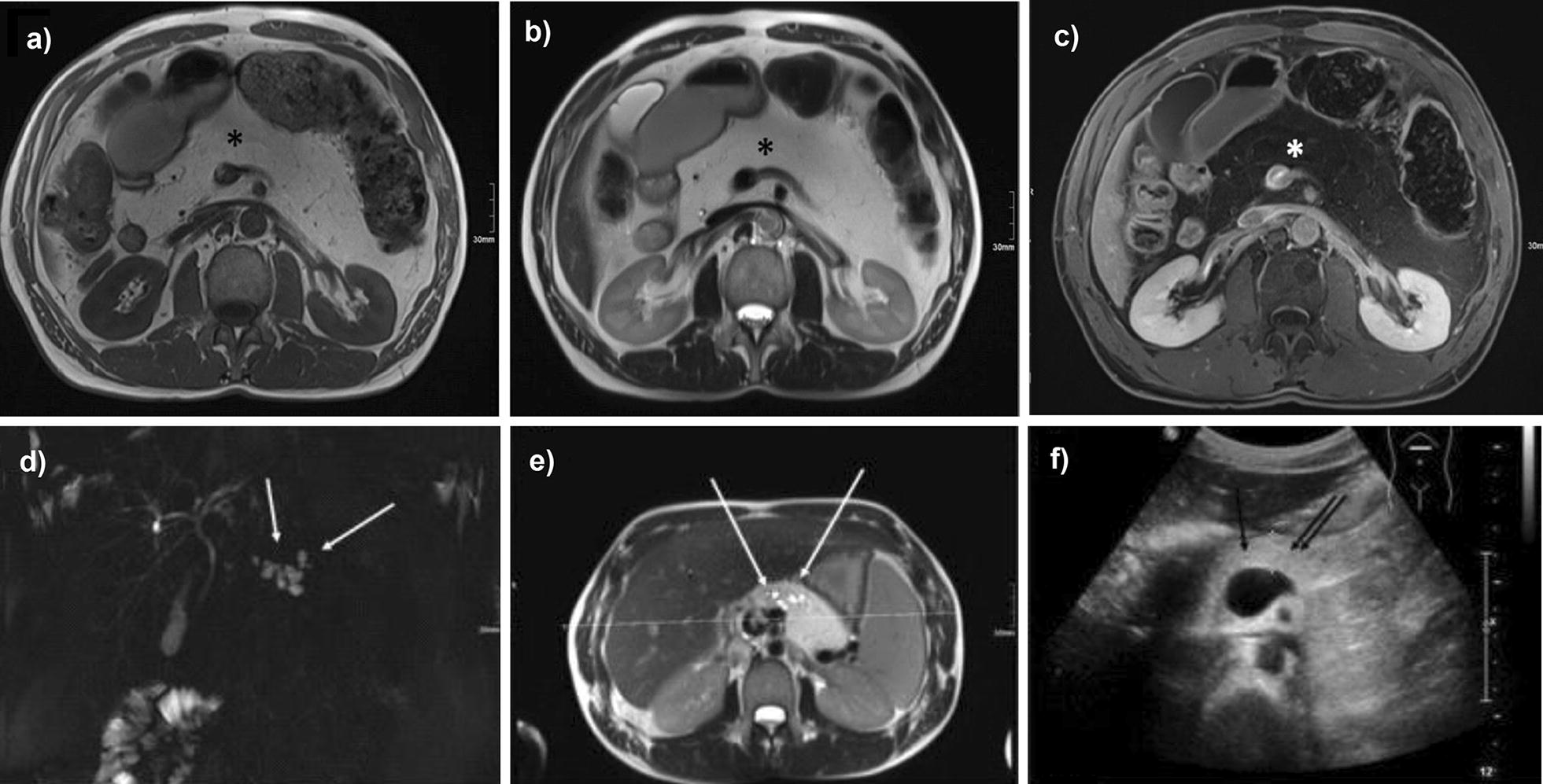
Transversal MRI images and a patient with manifold side branch cysts in pancreatic body. In all acquired sequences [T1-Flash-2D transversal native (**a**); T2-HASTE transversal (**b**); T1-Flash-2D fatsat transversal after contrast injection (**c**)] no residual pancreatic parenchyma can be identified. Asterixis mark empty pancreatic bed after full fatty transformation. Patient with manifold side branch cysts in pancreatic body with can be easily identified in T2-Space3D coronar MIP-MRCP sequence (**d**) and transversal T2-HASTE sequence (**e**). In B-mode sonography (**f**), identification of exact number, localization and contact to pancreatic duct was not possible

In 10/19 patients (52.6%) pancreatic parenchyma could not be identified by MRI because of complete lipomatous transformation, whereby pancreatic parenchyma could be detected by ultrasound (Fig. 2a–c). In contrast number and locations of cystic lesions were well identified in MRI imaging by 3-dimensional sequences in MRCP (Fig. 2d–f). In all patients also diffusion weighted imaging was performed, additional information was here only given for identification of cystic lesions. In 1/19 patients (5.2%) sonographic evaluation of pancreatic size was not applicable. Significant differences were registered for size of pancreatic body with a bigger diameter in MRI (1.4 ± 0.6 cm) than in ultrasound (1.0 ± 0.4 cm) (p = 0.049) (Table 1). There was also no statistical significance between the size of pancreatic duct and the size of cystic lesions between ultrasound and MRI (p = 0.157 respectively p = 0.999). An overview of measure signal intensity of evaluable pancreatic head by T1w and T2w MRI sequences is given on Additional file 1: Table S1.

**Table 1:** Evaluated parameters in both imaging and VTQ (in m/s) shear wave velocities

n = 19	Ultrasound	MRI	p-value
	Mean ± SD	Mean ± SD	
Caput size (cm)	1.89 ± 0.57	1.52 ± 0.76	0.858
Body size (cm)	1.02 ± 0.41	1.39 ± 0.64	0.049
Tail size (cm)	1.78 ±0.73	1.70 ± 0.40	0.461
Size pancreatic duct (mm)	0.78 ± 0.17	2.18 ± 0.26	0.157
Size of cystic lesion (mm)	10.83 ± 5.34	12.20 ± 10.05	0.655

	n (%)		p-value
Presentability of pancreatic duct	13/19 (68.4%)	8/19 (42.1%)	0.180
Chronic Inflammation	9/19 (47.4)	3/19 (15.8%)	0.109
Malignoma	0/19 (0.0%)	0/19 (0.0%)	
Cystic lesions	6/19 (31.6%)	5/19 (26.3%)	0.999

	Normal pancreatic parenchyma (n = 9)	Full fatty transformation of pancreas (n = 10)	p-value
	Mean ± SD	Mean ± SD	
Head of the pancreas	1.13 ± 0.39	1.18 ± 0.53	0.968
Body of the pancreas	1.01 ± 0.29	0.93 ± 0.23	0.657
Tail of the pancreas	1.12 ± 0.48	0.96 ± 0.23	0.840

Evaluated parameters in both imaging and VTQ (in m/s) shear wave velocities of the pancreas in patients with residual normal parenchyma vs. full fatty transformation of the parenchyma

#### Correlations of MRI and shear wave elastography

pSWE-values showed no significant differences between the two subgroups in all pancreatic parts (Table 1). pSWE-values of pancreatic parenchyma did not correlate with measured signal intensity values and also SI ratios, neither with T1w-GRE (head: p = 0.930, r = 0.025; p = 0.435, r = 0.218) nor with T2w HASTE sequences (head: p = 0.152, r = − 0.375; p = 0.860, r = − 0.050). No significant correlations to signal intensity-ratio pancreatic head/liver on T1w-falsh (p = 0.334, r = 0.268) and T2w HASTE sequences (p = 0.863, r = − 0.047) were registered. In the subgroups of patients with residual pancreas parenchyma and with full fatty transformation no significant correlations were detected, neither between pSWE and T1w (p = 0.420, r = 0.333), nor between pSWE and T2w (p = 0.067, r = − 0.633). Comparing full fatty transformation and residual pancreatic parenchyma, significant differences were seen in T1w (p = 0.045), not in T2w sequences (p = 0.080) (Additional file 1: Table S1).

### Discussion

To our knowledge, this is the first study comparing B-mode ultrasound including shear wave velocities of the pancreas with MRI imaging in patients with CF. We showed that ultrasound is superior to MRI in case of complete fibro-fatty transformation of the parenchyma and for evaluation of the pancreatic duct. pSWE did not correlate directly with measured intensities of pancreatic parenchyma in MRI, neither in the whole CF collective nor in subgroups of fatty transformation of the parenchyma.

Comparison of morphological aspects of the pancreas revealed that 10/19 patients showed a complete lipomatous transformation of the parenchyma, which can be identified more easily in MRI. Significant differences were registered for the diameter of the pancreatic body. This could be explained by suboptimal conditions in ultrasound examination due to different fasting periods and gas overlay. No significance were registered for pancreatic head and pancreatic tail, which demonstrates the comparability of both imaging methods, especially under consideration of the visibility of this challenging anatomic parts.

Current studies by Pandey et al. and [12] Jeon et al. [14] showed reproducible size measurements of pancreas in MRI, however, not in CF patients but in patients with intraductal papillary mucinous neoplasm (IPMN), non hypervascular pancreatic neuroendocrine tumor (PNET) and pancreatic ductal adenocarcinoma (PDAC).

The sonographically determined mean diameter of pancreatic head with 1.9 ± 0.6 cm is comparable to published results in literature with 2.1 ± 0.8 cm in CF patients, even when compared to data on healthy controls with 2.0 ± 0.5 cm [10]. In contrast to other ultrasound studies [15] our collective did not show anatomically altered or in diameter reduced pancreatic size.

When comparing the subgroups of full fatty transformation with residual pancreatic parenchyma no differences in pSWE could be registered. VTQ shear wave velocities were comparable to prior studies by Yashima et al [16]. Former studies did already show accuracy of shear wave elastography in pancreatic parenchyma, especially after phases of pancreatitis, however in otherwise healthy patients [17, 18]. Perhaps in our subgroup of residual parenchyma the tissue is softer than in regular persons, because of earlier pancreatitis [10, 19]. Shear wave elastography in patients with CF were only used in the past for analyzing associated liver disease [20].

MRI signal intensity did not correlate with pSWE-values on T1w-flash and T2w HASTE sequences. Even pSWE-values of pancreatic head did not correlate with measured signal intensity-ratio pancreatic head/muscle erector spine or intensity-ratio pancreatic head/liver parenchyma. In the subgroups of patients with residual pancreas parenchyma and with a complete fatty transformation no significant correlations can be registered between pSWE and T1w and T2w. Generally the values of MRI imaging in CF patients, especially in pancreas were established before since the early 90s [21–23]. A possible restriction of our correlations was the ratio of signal intensity of pancreatic parenchyma to the liver, because of theoretically involvement of this organ into CF manifestations. Nevertheless even the more objective ratio with muscle did not show significant differences. In all examinations the same MRI scanner with the same parameters was used, which allowed comparability between patients. Comparisons between shear wave elastography and MRI are currently only established in musculoskeletal imaging in patients with delayed onset muscle soreness, however, also with superiority of ultrasound to MR-imaging [24].

In conclusion, MRI imaging corresponded with ultrasound in our study in case of morphological aspects, but did not correlate with pSWE. For visualization of cystic lesions and fatty transformation MRI imaging is superior to conventional ultrasound.

## Limitations

Our work has certain limitations. First, only a small number of patients was evaluated by the study because of the rarity of CF. Second, acquisition of ultrasound examination was performed not by the reader of the MRI images. Third, the study is a single centre study. Overall this projected should be extended to a larger cohort. A larger patient cohort would be the most helpful benefit for further studies, for example by a multi-centre study. This would be helpful because of the rarity of cystic fibrosis.

## Additional file

**Additional file 1: Table S1.** Overview of measure signal intensity of pancreatic head by T1w and T2w MRI sequences.

## Abbreviations

CF: cystic fibrosis
pSWE: point shear wave elastography
MRCP: MR-cholangiopancreatography
DWI: diffusion weighted imaging
VTQ: virtual touch quantification
SI: signal intensities
BMI: body mass index
IPMN: intraductal papillary mucinous neoplasm
PDAC: pancreatic ductal adenocarcinoma
Ca 19-9: carbohydrate antigen 19-9
CEA: carcinoembryonic antigen
