# Supplementary material for: Diagnostic accuracy of MRI with MRCP and B-Mode-sonography with elastography of the pancreas in patients with cystic fibrosis: a point-to-point comparison
Source: BMC Res Notes. 2019 Mar 18;12:150. doi: 10.1186/s13104-019-4193-4 (PMC6423810; doi:10.1186/s13104-019-4193-4)
Supplement: Supplementary file 1 — Additional file 1: Table S1. Overview of measure signal intensity of pancreatic head by T1w and T2w MRI sequences. [file 13104_2019_4193_MOESM1_ESM.docx]

**Additional file: Table S1**

**Table S1:** Overview of measure signal intensity of pancreatic head by T1w and T2w MRI sequences.

|  | **Mean ± SD** | | | |
| --- | --- | --- | --- | --- |
|  | **T1w** | **Area** | **T2w** | **Area** |
| **pancreas** | 307.53 ± 126.62 | 20.78 ± 12.90 | 570.56 ± 259.71 | 26.25 ± 20.65 |
| **muscle** | 177.40 ± 38.97 | 26.53 ± 22.18 | 138.32 ± 47.89 | 30.98 ± 40.26 |
| **liver** | 261.73 ± 50.64 | 28.98 ± 17.87 | 266.79 ± 77.51 | 31.64 ± 20.54 |
| **Ratio pancreas/muscle** | 1.88 ± 0.84 |  | 8.86 ± 19.31 |  |
| **Ratio pancreas/liver** | 1.21 ± 0.55 |  | 3.58 ± 6.06 |  |

Area is measured in mm^2^
